# RMDGCN: Prediction of RNA methylation and disease associations based on graph convolutional network with attention mechanism

**DOI:** 10.1371/journal.pcbi.1011677

**Published:** 2023-12-06

**Authors:** Lian Liu, Yumeng Zhou, Xiujuan Lei

**Affiliations:** School of Computer Science, Shaanxi Normal University, Xi’an, Shaanxi, China; University of California Riverside, UNITED STATES

## Abstract

RNA modification is a post transcriptional modification that occurs in all organisms and plays a crucial role in the stages of RNA life, closely related to many life processes. As one of the newly discovered modifications, N1-methyladenosine (m^1^A) plays an important role in gene expression regulation, closely related to the occurrence and development of diseases. However, due to the low abundance of m^1^A, verifying the associations between m^1^As and diseases through wet experiments requires a great quantity of manpower and resources. In this study, we proposed a computational method for predicting the associations of **R**NA **m**ethylation and **d**isease based on **g**raph **c**onvolutional **n**etwork (RMDGCN) with attention mechanism. We build an adjacency matrix through the collected m^1^As and diseases associations, and use positive-unlabeled learning to increase the number of positive samples. By extracting the features of m^1^As and diseases, a heterogeneous network is constructed, and a GCN with attention mechanism is adopted to predict the associations between m^1^As and diseases. The experimental results indicate that under a 5-fold cross validation, RMDGCN is superior to other methods (AUC = 0.9892 and AUPR = 0.8682). In addition, case studies indicate that RMDGCN can predict the relationships between unknown m^1^As and diseases. In summary, RMDGCN is an effective method for predicting the associations between m^1^As and diseases.

## Author summary

- As a new epitranscriptomic modification, m^1^A plays an important role in the gene expression regulation, closely related to the occurrence and development of diseases.
- However, due to the low abundance of m^1^A, verifying the associations between m^1^As and diseases through wet experiments requires a great quantity of manpower and resources.
- It is especially important to develop computational methods for predicting the associations between m^1^A modifications and diseases.
- We developed a deep learning model to predict the associations of m^1^As and diseases, namely RMDGCN.
- RMDGCN increases the number of known relationships between m^1^As and diseases through PU learning, and combines m^1^A similarity network and disease similarity network to construct heterogeneous networks. It adopts GCN with layered attention mechanism to predict the associations between methylations and diseases.
- The results of the 5-fold cross validation show that the performance of RMDGCN is superior to other comparison algorithms.
- Through case study analysis of breast cancer, RMDGCN can effectively predict the relationships between unknown m^1^As and diseases.

## Introduction

RNA epigenetic modifications occur in almost all types of RNA and play an important role in all stages of life. Up to now, more than 170 different RNA modifications have been identified. As one of the modifications, N1-methyladenosine (m^1^A) is a new epigenetic one, and the nitrogen in the first position of adenine is modified by a methyl group during the process [1]. The University of Chicago analyzed the methylation of m^1^A in eukaryotic mRNA by m^1^A RNA methylation sequencing and RIP sequencing techniques, and the appearance of the m^1^A chemical modification was to significantly enhance protein translation of transcripts. It was further observed that m^1^A modification is prevalent in humans, rodents and yeast, which suggested that m^1^A modification is evolutionarily conserved [2]. With the development of high-throughput sequencing technologies, several studies have identified methylation sites for m^1^A in nuclear and mitochondrial RNA. The results show that unlike the prevalent m^6^A modification, the abundance of m^1^A is relatively low and most of the m^1^A methylation modification sites are concentrated in the 5’UTR of the mRNA transcript, particularly at the first and second positions of the transcript initiation site. A small proportion of sequences were created by a known methylation enzyme complex, TRMT6 / 61A, conform to the ’GUUCRA’ sequence motif [3]. A large number of m^1^A methylation modifications were found in the mitochondrially encoded transcripts by using m^1^A mapping techniques. Among them, m^1^A within the 5’UTR of mRNA transcripts can promote protein translation, while m^1^A observed in the coding region of transcripts can lead to inhibition of translation. In addition, ALKBH1, an RNA repair enzyme, can function as an m^1^A scavenger by catalyzing the demethylation reaction [4]. And m^1^A can affect ribosome biosynthesis and mediate antibiotic resistance in rRNA [5], and mediate tRNA responses to environmental stress [6,7].

Some researchers have shown that genetic variants function can alter RNA modification by specifically replacing nucleotides at the modification site or altering the nucleotide sequence in the proximal flanking region [8]. More and more scientific studies have shown that m^1^A methylation sites are closely associated with human diseases, such as cardiovascular disease [9], cancer [10], *etc*. Due to the high cost and complexity of wet experiments used to verify the associations between m^1^A modification sites and diseases, predicting the associations between m^1^A sites and diseases based on computational methods can help predict potential relationships between m^1^As and diseases. Therefore, it is necessary to develop effective methods for predicting the associations between m^1^A sites and diseases. The associations prediction between m^1^A modification sites and diseases is a computational approach to predict the relationships between unknown m^1^A modification sites and diseases based on experimentally confirmed m^1^A modification sites and diseases associations. So far, there has been no specific method for predicting the associations between m^1^A sites and diseases. However, there are some methods for predicting the associations between N7-methylguanosine and diseases. m7GDisAI [11] integrates the location information of m^7^G and the comprehensive similarity information of diseases to construct a heterogeneous network, on which matrix decomposition methods are applied to predict potential disease-related m^7^G sites. HN-CNN [12] constructs heterogeneous networks based on m^7^G site similarity, disease similarity, and the associations between m^7^G and diseases to form features of m^7^G site-disease pairs. Then, multidimensional and uncorrelated features are obtained through convolutional neural networks (CNN) [13]. And XGBoost was used to predict the correlation between m^7^G site and disease. BRPCA [14] can accelerate the presence of noise and redundancy in association and similar information. And by introducing appropriate bounded constraints, it ensures that the predicted correlation score is within a meaningful interval. Numerous experiments have demonstrated the superiority and robustness of BRPCA. m7GDP-RW [15] combines the m^7^G sites and diseases feature information with known m^7^G-disease associations to calculate m^7^G similarity and disease similarity. Then, a heterogeneous network of m^7^G- diseases is constructed by combining the known m^7^G-disease associations with the computational similarity of m^7^G and diseases. Finally, a two times random walk algorithm with restart is used to search for new m^7^G-disease associations on the heterogeneous network.

Although there are few cases to solve the problem of predicting RNA methylation modification sites and diseases associations, various models developed in the past few years have made great progress in predicting potential miRNA-disease associations, lncRNA-disease associations, drug targets, *etc*. The existing methods can be roughly divided into two categories: network-based methods and machine learning-based algorithms.

The network-based algorithm constructs the associated data into a network and uses the model on the network to solve the problem. Tang *et al*. [16] proposed a double Laplacian regularization matrix completion model for miRNA-disease associations prediction, which transformed miRNA-disease associations prediction into a matrix complementation problem, using double Laplacian regularization terms to make full use of miRNA functional similarity and disease semantic similarity for miRNA-disease associations matrix complementation. The AUC of GLOOCV and LLOOCV were 0.9174 and 0.8289, respectively. Gu *et al*. [17] developed a global network random walk model to predict the potential lncRNA-disease associations GrwLDA, which can be used for disease with unknown associated lncRNA (isolated disease) and lncRNA with unknown associated disease (novel lncRNA). Wang *et al*. [18] proposed a weighted graph regularization collaborative non-negative matrix decomposition model to reconstruct the associations adjacency matrix between drugs and diseases by using weighted k-nearest neighbors, and to identify potential associations between drugs and diseases by using a graph regularization non-negative matrix decomposition model.

The machine learning-based approach treats the associations between histological data and diseases as a triadic set of samples, and models the histological data-disease associations problem as a classification problem on the sample set, which is classified by machine learning methods. Chen *et al*. [19] proposed a Kronecker regularized least squares method based on multiple kernel learning for miRNA-disease associations prediction, introducing Kronecker regularized least squares, which can reveal potential miRNA-disease associations by automatically optimizing the combination of multiple kernels of diseases and miRNAs. Zhang *et al*. [20] proposed a similar constraint matrix decomposition method for drug-disease associations prediction SCMFDD, which considers the biological background of the problem and uses known drug-disease associations, the drug features and diseases semantic information to project the drug-disease associations into two low-order spaces that reveal the underlying features of the drugs and diseases. Peng *et al*. [21] proposed to use neural networks for learning based miRNA-disease associations recognition framework, and to construct a three-layer heterogeneous network based on the disease similarity network, miRNA similarity network and protein interaction network to extract association features, and to use automatic encoder for feature dimension reduction. The features after dimension reduction are input into CNN, after which CNN completes the prediction task.

Traditional convolutional neural networks have brought great improvements in the field of text and image processing, but it can only process Euclidean spatial data. Due to the prevalence of graph data, researchers started to focus on how to construct deep learning models on graphs. Bruna *et al*. [22] first proposed Graph Convolution Network (GCN) and defined graph convolution in spectral space based on convolution theorem. CRPGCN [23] proposed a GCN constructed based on restart random walk (RWR) and principal component analysis (PCA) to predict the associations between circRNA and diseases. The RWR algorithm is used to improve the similarity associations between nodes and their neighbors and the PCA method performs dimension reduction and feature extraction. The GCN algorithm learns the features between circRNAs and the diseases and calculates the final similarity score. Hou *et al*. [24] used two GCNs (Asso-GCN and Sim-GCN) to extract features of piRNAs and diseases by obtaining association patterns from piRNA-disease interaction networks and two similar networks. GCN captures complex network structure information from the networks and learns to identify features, using fully connected networks and internal yield as output modules to predict piRNA-disease associations scores. Zhang *et al*. [25] proposed a lncRNA-disease associations prediction model SGALDA based on a semantic and global dual attention mechanism, which divides the constructed heterogeneous network into multiple sub-networks and applies the GCN on each sub-network separately to extract the semantic features of nodes to capture the higher-order interactions on the heterogeneous network.

In this study, we proposed RMDGCN to predict the associations between m^1^As and diseases based on positive-unlabeled learning (PU learning) and GCN. We obtain the similarity matrix of m^1^A sites through cosine similarity calculation, and the similarity matrix of diseases through disease symptom similarity and semantic similarity. They form a heterogeneous network with the adjacency matrix by PU learning. Finally, a GCN with attention mechanism is applied to the heterogeneous network to predict potential m^1^A modification sites related to diseases. In order to verify the effectiveness of RMDGCN, we used a 5-fold cross validation method to compare this method with other methods. The experimental results show that RMDGCN results are significantly superior to other methods and can well predict the potential m^1^A sites associated with disease.

## Results and discussion

### Evaluation metrics

In order to evaluate the performance of the model, we use 5-fold cross validation (5CV) to verify. In the 5CV, all known associations between m^1^As and diseases were randomly divided into 5 parts, each part is considered as a test set, and the remaining four parts are considered as training sets. In the test set, we set the known associations between m^1^As and diseases in the $\tilde{A}$ matrix to 0, and then obtain the predicted scores by RMDGCN. Then the prediction scores are sorted in descending order. We drew the receiver operating characteristic (ROC) curve and the Precision-Recall (PR) curve, and calculated the area under the ROC curve and the area under the PR curve (AUPR) to evaluate the model performance. The ROC curve is obtained by *TPR* and *FPR* under different scoring thresholds, and the PR curve is obtained by *precision* and *recall* under different scoring thresholds. *TPR*, *FPR*, *precision* and *recall* are calculated by Eq 1. The AUC is not sensitive to whether the sample category is balanced. The performance under the condition of highly unbalanced data is still too ideal to show the actual situation well. Under extremely unbalanced data (fewer positive samples), PR curve may be more practical than ROC curve. We use AUC and AUPR as the main evaluation indicators.

In addition, we adopt *Precision*, *Recall*, Accuracy (*ACC*) and *F*1*_score* to show the results of the model, which are defined as follows respectively:

$$\begin{array}{l} TPR=\frac{TP}{TP+FN}\\ FPR=\frac{FP}{FP+TN}\\ Precision=\frac{TP}{TP+FP}\\ Recall=\frac{TP}{TP+FN}\\ ACC=\frac{TP+TN}{TN+FP+TP+FN}\\ F1\_score=\frac{2\times Precision\times Recall}{Precision+Recall} \end{array}$$ (1)

where *TP* is true positive, *TN* is true negative, *FP* is false positive, and *FN* is false negative.

### Adjustment of parameters

**Fig 1:**
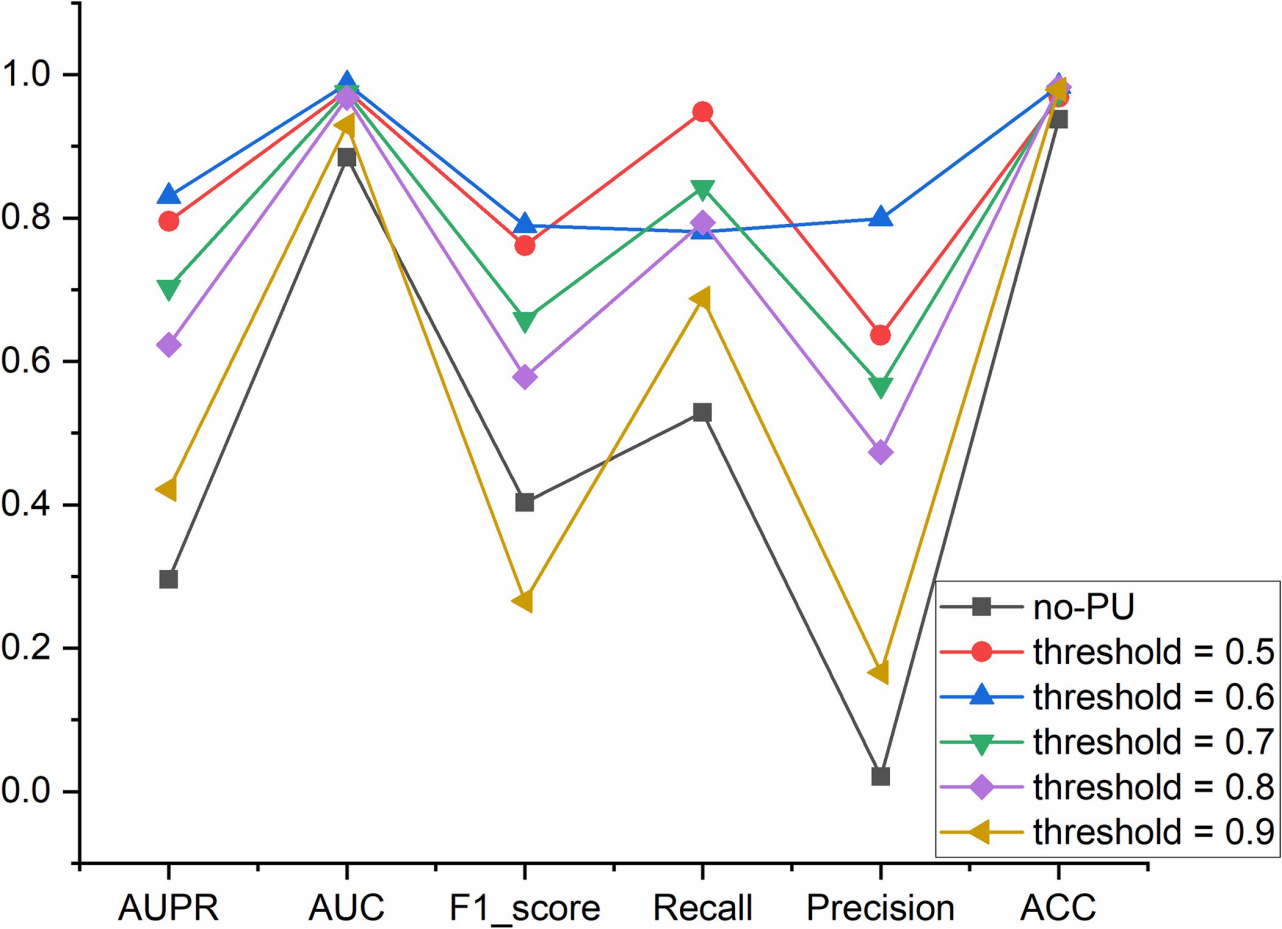
The results of the different *thresholds*.

In RMDGCN, many parameters need to be adjusted, including PU learning *threshold*, similarity parameter *λ*, penalty factor *α* and *β*, and the embedding dimension. The purpose of PU learning is to obtain more positive samples, but PU learning only obtains predictive scores for the relationships between m^1^A modification sites and diseases. In order to obtain the corresponding adjacency matrix, it is necessary to obtain a 0–1 relationship matrix based on the set threshold. In general, we set the classification threshold to 0.5. In order to obtain higher confidence results, we set the *threshold* = [0.5,0.6,0.7,0.8,0.9]. Then, we compared the results of learning with PU learning and without PU learning, and the results of PU learning were achieved through different *thresholds*. When not performing PU learning, only the standard adjacency matrix *A* obtained from the beginning is used. When performing PU learning, a new adjacency matrix $\tilde{A}$ is obtained based on the set threshold. If the score of PU learning is greater than the *threshold*, it is 1, otherwise it is 0. From Fig 1, it can be seen that the results of learning with PU learning are significantly better than those without PU learning. When *threshold* = 0.6, all results are optimal except for *Recall*. Therefore, we set the threshold to 0.6. When *threshold* = 0.6, the positive samples (i.e. $\tilde{A_{\mathrm{ij}}}$ = 1) in the adjacency matrix $\tilde{A}$ are 11034.

**Fig 2:**
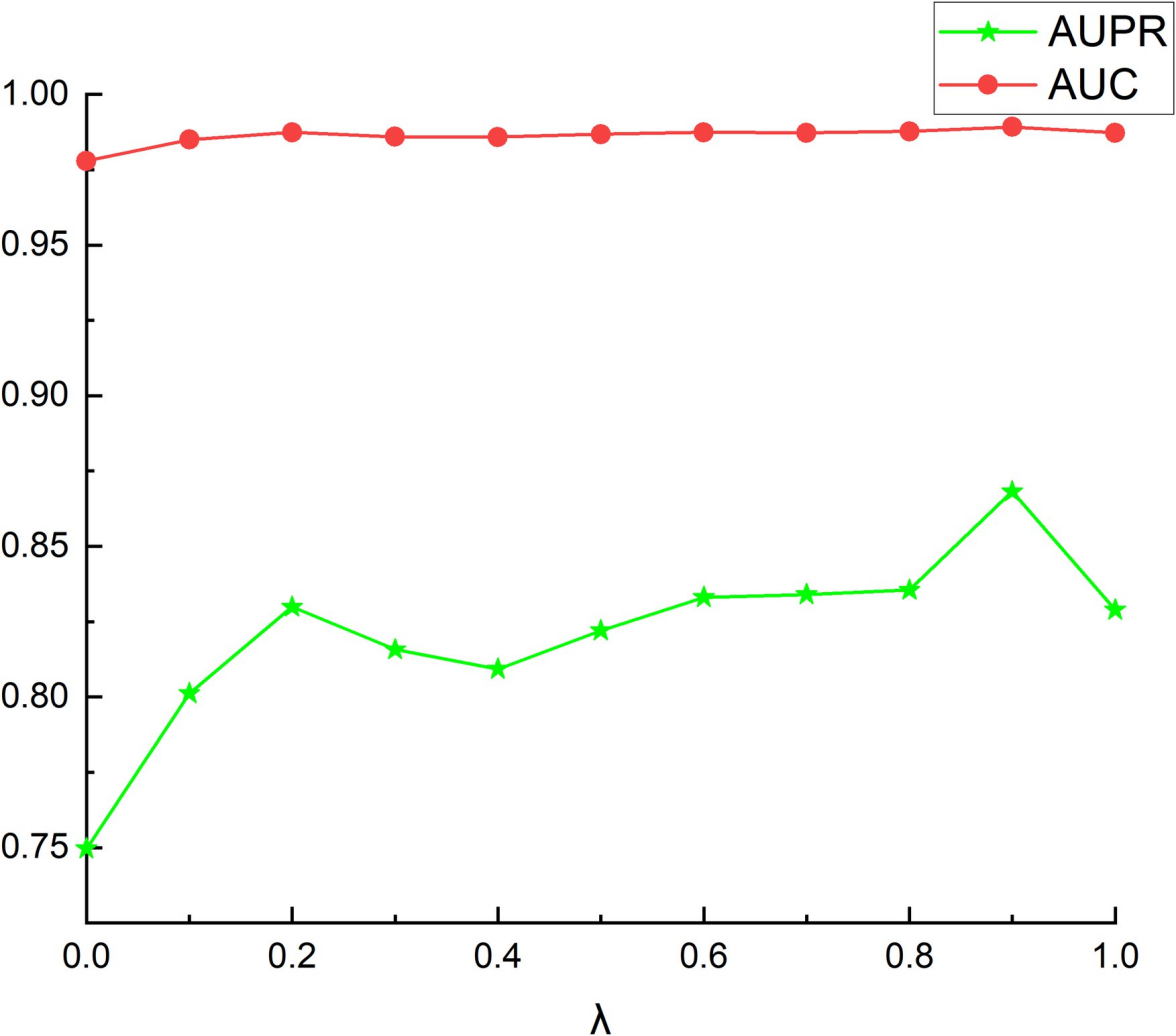
The AUCs and AUPRs between different *λ*.

Then we discussed the disease feature fusion parameter *λ*, and set *λ* = [0,0.1,0.2,0.3,0.4,0.5,0.6,0.7,0.8,0.9,1]. When *λ* = 0, disease similarity features are only composed of semantic similarity, and when *λ* = 1, disease similarity features are composed of symptom similarity. From Fig 2, it can be seen that when *λ* = 0.9, AUC and AUPR can achieve the maximum value, and the performance of the whole model is optimal. This shows that the symptom characteristics of diseases play a more important role in building heterogeneous networks using disease similarity features.

**Fig 3:**
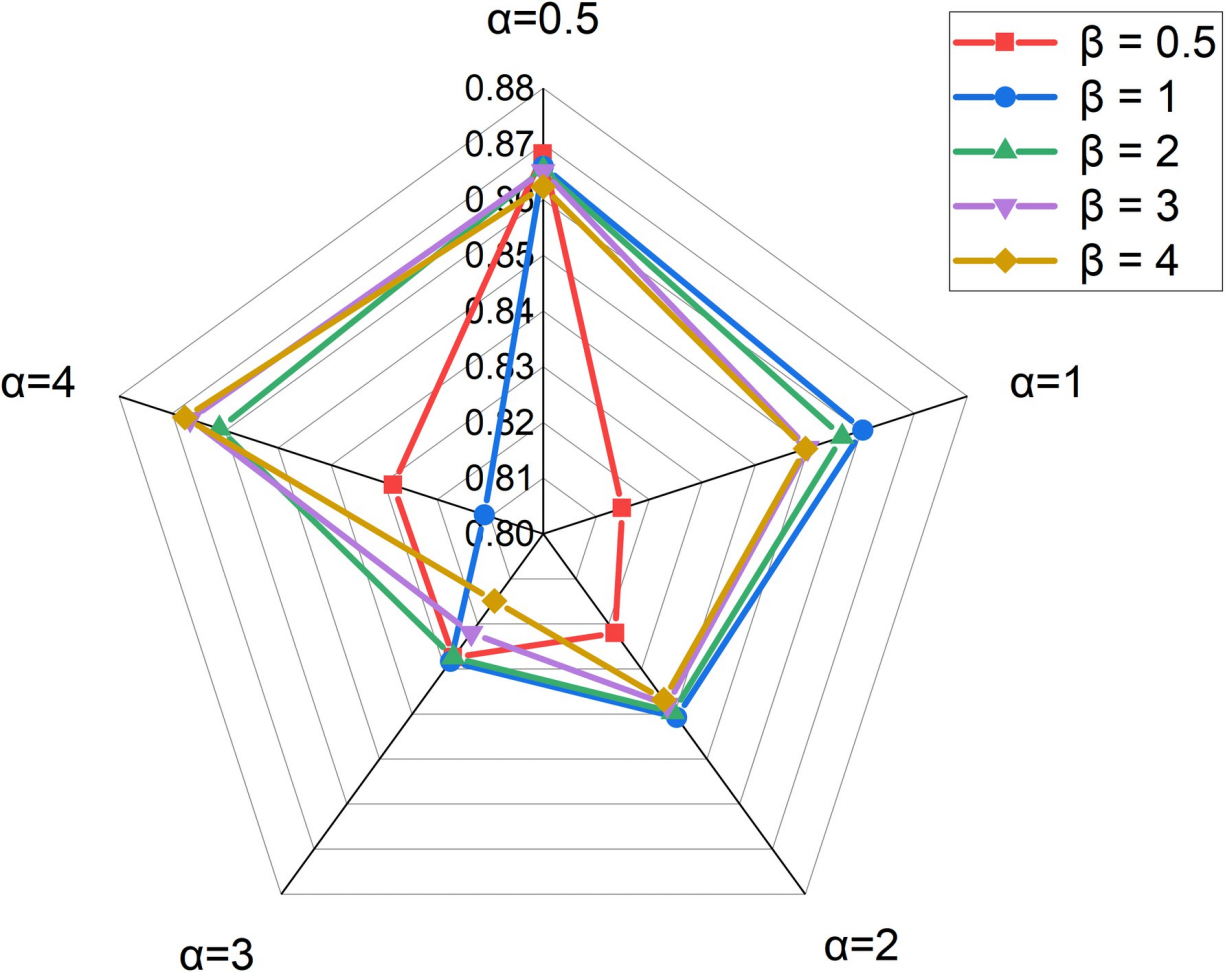
The AUPRs results of *α* and *β*.

Penalty factors *α* and *β* are mainly used to control the contribution of similarity in GCN propagation. We let *α* and *β* both as [0.5,1,2,3,4]. Because the AUCs are not very different, we only show the AUPRs. As a result, RMDGCN (*α* = 0.5 and *β* = 0.5) gained the highest AUPR of 0.8682 in 5CV as shown in Fig 3.

**Fig 4:**
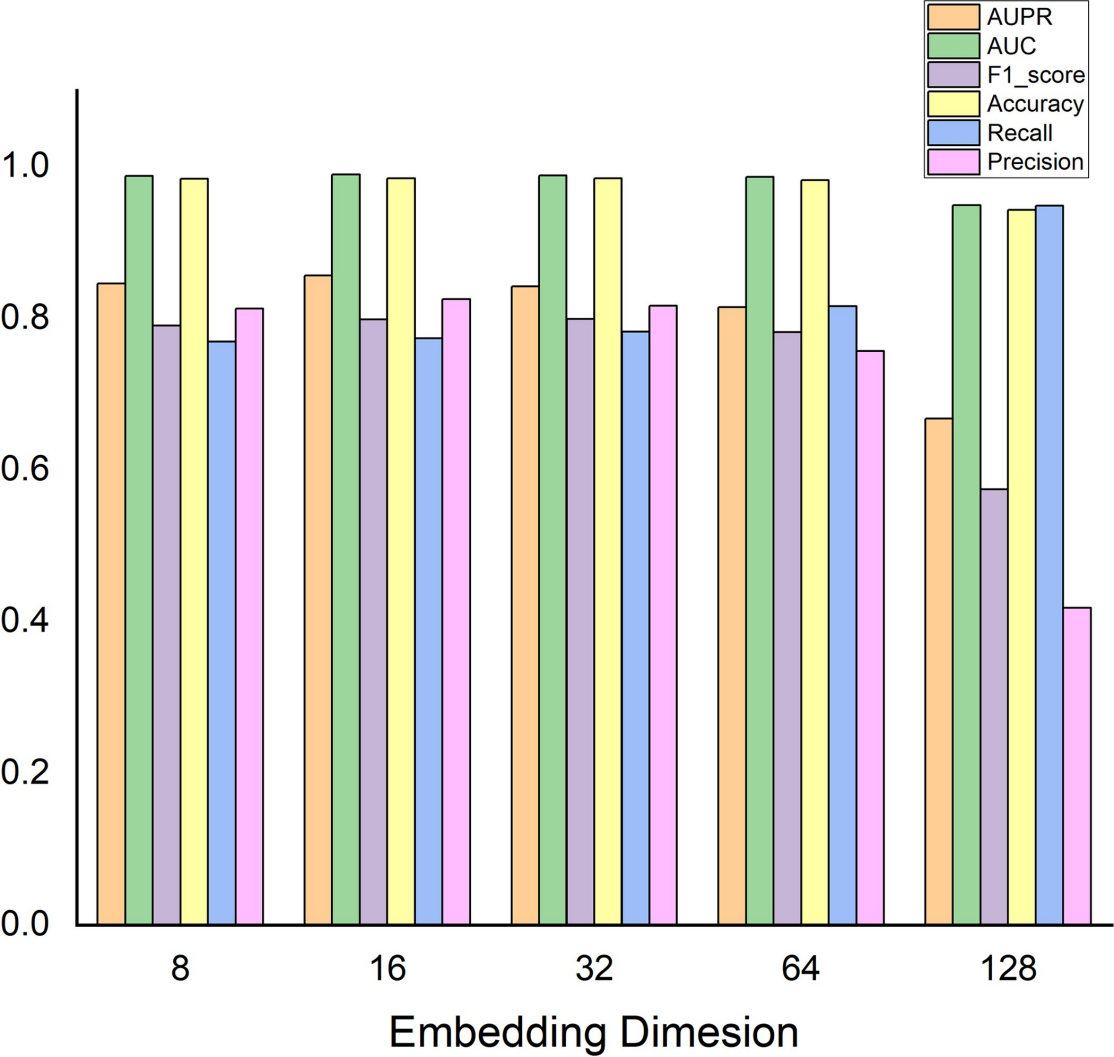
The results of embedding dimension.

Finally, we analyzed the impact of embedding dimensions on the experimental results. We set the embedding dimension as [8,16,32,64,128], and the experimental results (see Fig 4) show that the optimal experimental results can be obtained when the embedding dimension is 16.

### Comparing with other methods

In order to analyze the performance of RMDGCN in predicting the relationships between m^1^A modification sites and diseases, we compared it with the commonly used methods of relationship prediction, RWR and non-negative matrix factorization (NMF), under the five-fold cross-validation. The RWR and NMF used here are basic models without any improvement. In addition, we also compared RMDGCN with existing m^7^G-disease associations prediction method BRPCA [14], drug and disease prediction methods SCMDDF [20], and miRNA-disease associations prediction method LOMDA [26]. RWR can use the multi-faceted information of nodes to obtain the correlation score between nodes by capturing the global information of the graph. Since the same molecule may cause many different diseases, we believe that the potential factors controlling the molecular disease associations are highly correlated. NMF decomposes the association matrix into base matrix and weight matrix. The base matrix describes the relative contribution of potential regulatory factors, and the weight matrix describes the relative contribution of diseases. The predicted score of the relationships between the modified sites and the diseases is obtained by multiplying the two matrices. BRPCA performs singular value decomposition on heterogeneous networks to recover missing items in the adjacency matrix of heterogeneous networks. SCMDDF is a method of using similarity constraint matrix factorization to predict the relationships between drugs and diseases, while LOMDA is a linear prediction method based on linear optimization methods for predicting the associations between miRNA and diseases.

**Fig 5:**
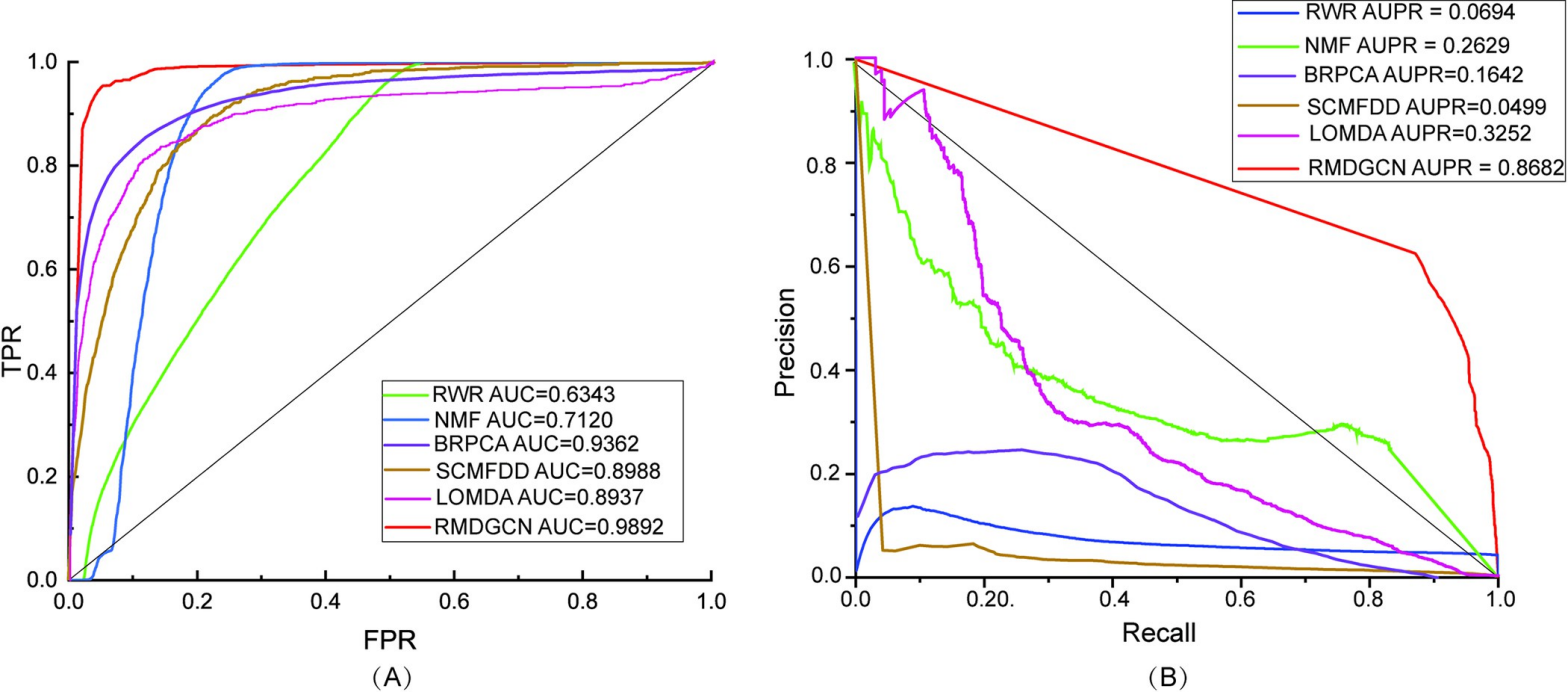
The ROC curves and PR curves for 5CV. (A) ROC curves (B) PR curves.

The ROC curves and PR curves of the several methods are shown in the Fig 5. It can be seen from the figure that RMDGCN performs best in predicting the relationships between m^1^A modification sites and diseases, which is 0.053 higher than the AUC of BRPCA, 0.0904 higher than SCMFDD, and 0.0955 higher than LOMDA. In addition, AUPR is 0.704 higher than BRPCA and 0.543 higher than LOMDA. Although the *ACC* of RMDGCN is lower than NMF and SCMFDD, and the *Recall* is lower than LOMFDA and BRPCA, all indicators of RMDGCN are relatively balanced and high. It proves that the performance of RMDGCN is superior to other methods. The detailed results of all evaluation indicators are shown in Table 1.

**Table 1:** Performance comparison of different methods.

	*AUC*	*AUPR*	*Precision*	*ACC*	*Recall*	*F*1*_score*
RWR	0.6343	0.0694	0.1041	0.9036	0.1900	0.1345
NMF	0.7120	0.2926	0.1038	**0.9949**	0.0843	0.0754
BRPCA	0.9362	0.1624	0.0579	0.9304	0.7847	0.1079
SCMFDD	0.8988	0.0499	0.0582	0.9915	0.162	0.0856
LOMDA	0.8937	0.3252	0.0172	0.3540	**0.9431**	0.0338
**RMDGCN**	**0.9892**	**0.8682**	**0.799**	0.9836	0.7809	**0.7897**

### Case study

In order to further verify the performance of the model, RMDGCN was used to predict the potential m^1^A sites associated with breast cancer, which is a disease with a very high prevalence rate among women worldwide. We further performed gene ontology (GO) analysis on the host genes of these predicted new sites.

In case study, all known associations between the breast cancer and m^1^As were assumed to be unknown. Then the prediction scores were calculated by RMDGCN between the breast cancer and m^1^A modifications. According to the prediction scores, the candidate m^1^A sites related to breast cancer will be sorted, and the top 500 candidate sites will be selected, and their host genes will be found. Because some sites have common host genes, we finally obtained 174 host genes for GO analysis, then, by removing redundancy, we obtained 150 host genes with unique gene symbols. GO analysis includes cell composition (CC), biological process (BP) and molecular function (MF). Then, DAVID describes these host genes from these three aspects. Among them, the p-value cutoff is set to 0.05. In order to control the error detection rate, the adjusted p-value is set to 0.1. When this condition is met, it indicates that the correlation between host gene and GO terms is statistically significant.

**Fig 6:**
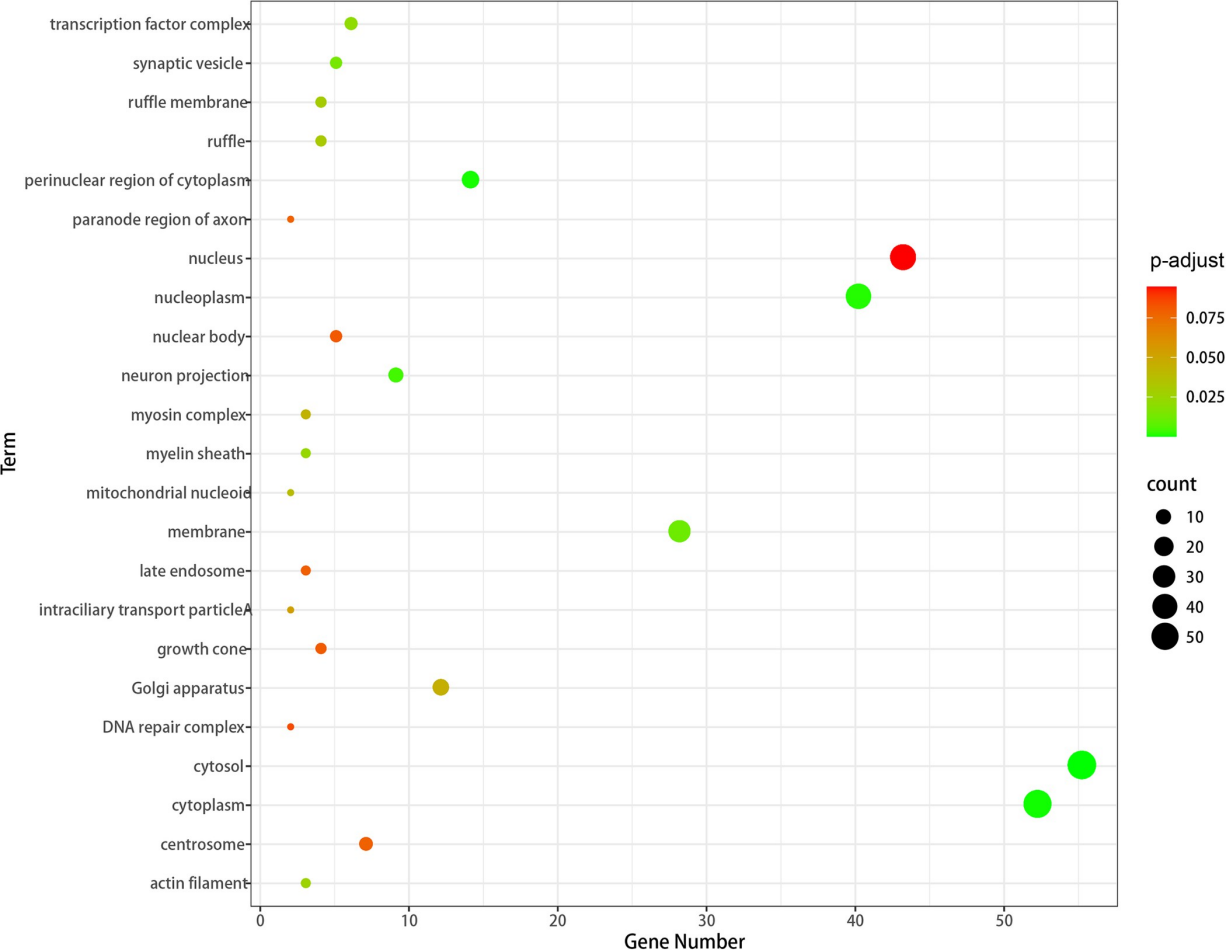
The CC enrichment term of genes related to breast cancer.

Ana *et al*. [27] showed that nucleolar protein was overexpressed in breast tumors, mainly in the nucleus, but cytoplasmic staining was observed in some cells. The CC enrichment term of genes related to breast cancer is shown in the Fig 6. The larger the circle, the richer the gene in this term. It can be seen that many genes related to breast cancer disease are enriched in the nucleus and cytoplasm, indicating that these sites may participate in the formation of nucleus and cytoplasm.

**Fig 7:**
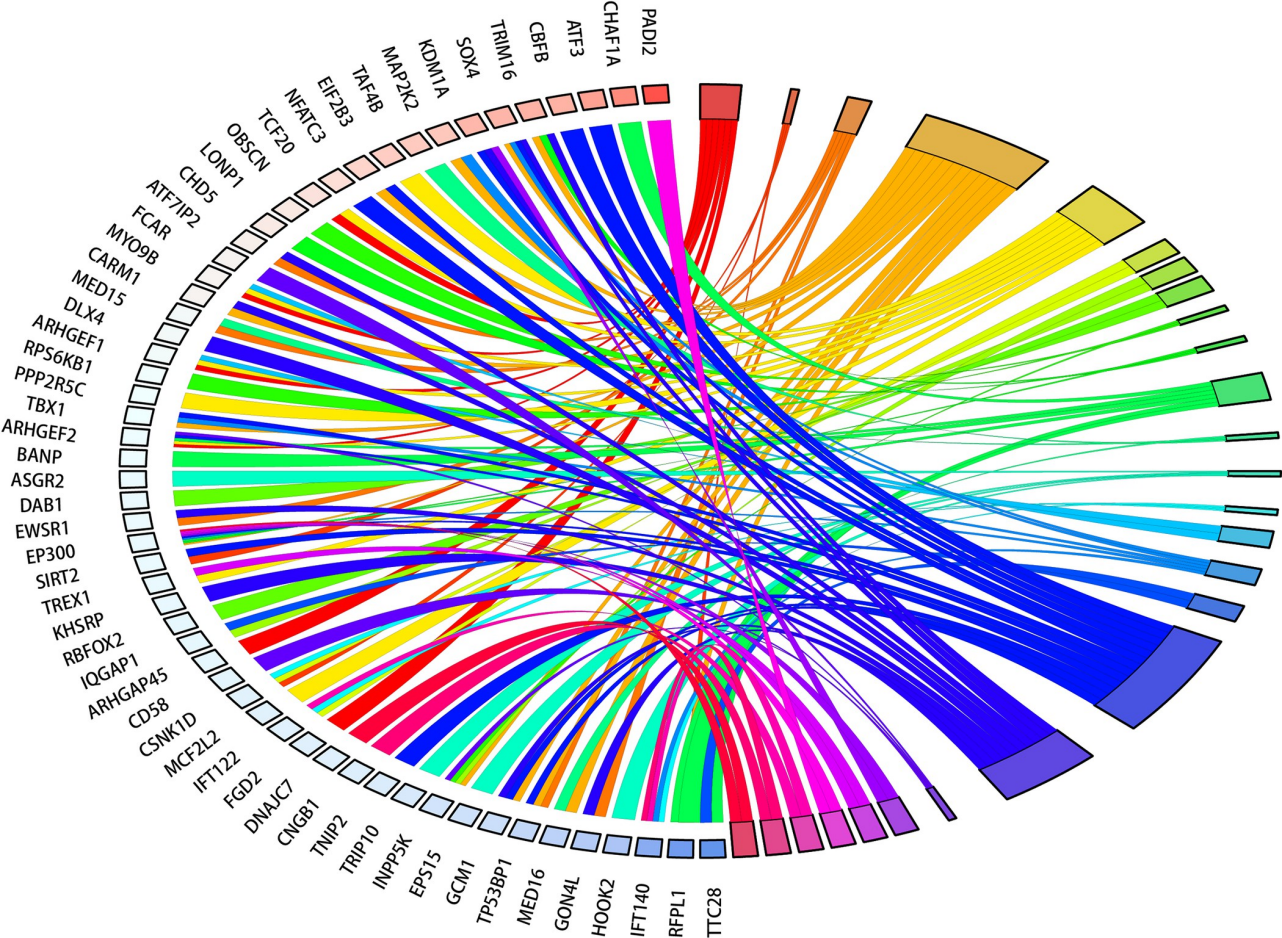
The relationship between the BP terms and the enriched host genes.

Research shows that the occurrence of breast cancer is related to a variety of biological processes. We analyzed the relationship between the BP terms and the enriched host genes, and the results are shown in the Fig 7. It can be seen from the figure that a gene may participate in multiple biological processes, and a biological process is the result of the interaction of multiple genes. For example, CHD5 [28] gene can inhibit the proliferation of breast cancer cells in vitro and slow down the process of cell cycle. Missense mutation in LONP1 and gene mutation in TRMT61B [29] are related to m^1^A modification level in a large number of tissues. The genetic variation related to the level of RNA modification is related to a variety of disease related phenotypes, including blood pressure, breast cancer and psoriasis, suggesting the role of mitochondrial RNA modification in complex diseases.

**Fig 8:**
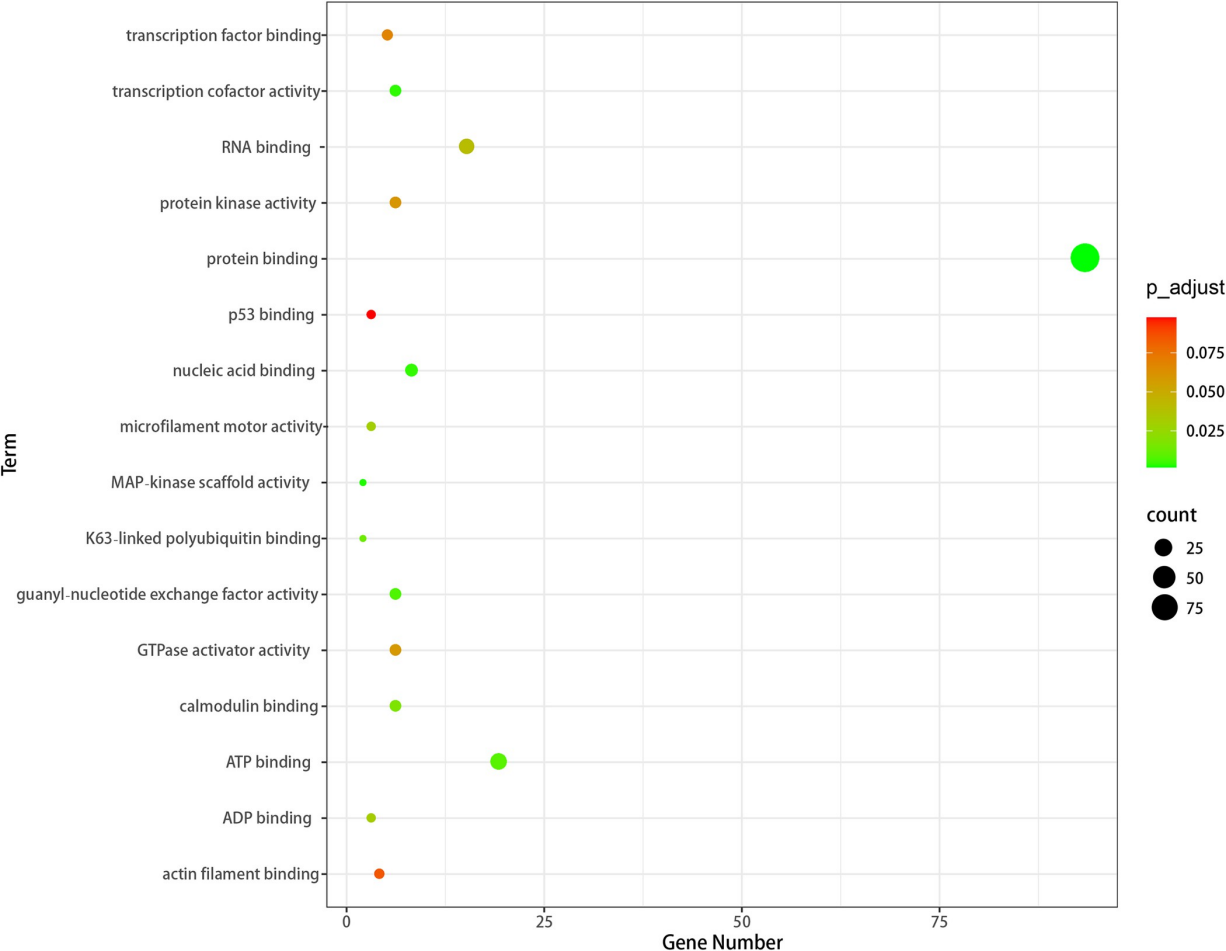
The MF enrichment term of genes related to breast cancer.

Next, we analyzed the MF enriched by GO, and the results are shown in the figure. It can be seen from Fig 8 that GO is mainly enriched in protein binding on MF. For example, p53 binding plays an important role in the repair of radiation-induced DNA double strand breaks (DSBs) [30]. As a tumor suppressor, it has a far-reaching effect on inhibiting breast cancer, and has become an important new biomarker to judge the prognosis of breast cancer.

## Conclusion

In this study, a RMDGCN method was developed to predict the associations between the m^1^A sites and various diseases, aiming to reveal the regulatory pathways of diseases regulated by the epitranscriptome layer. Using disease related mutations as links, the associations between m^1^A sites and diseases were extracted, and a m^1^A-disease associations network was constructed. In order to further improve the accuracy of prediction, PU learning is used to increase the number of associations.

RMDGCN is conducted on a heterogeneous network of m^1^A-disease associations, which is composed of m^1^A similarity network, disease similarity network, and m^1^A-disease associations network. In terms of predictive performance, the AUC of RMDGCN in 5CVs is as high as 0.9892, and the AUPR is as high as 0.8682. In addition, in the case study of breast cancer, we verified the effectiveness of RMDGCN through GO analysis. In summary, RMDGCN is a powerful tool for predicting the associations between methylation modification sites and diseases.

## Material and methods

**Fig 9:**
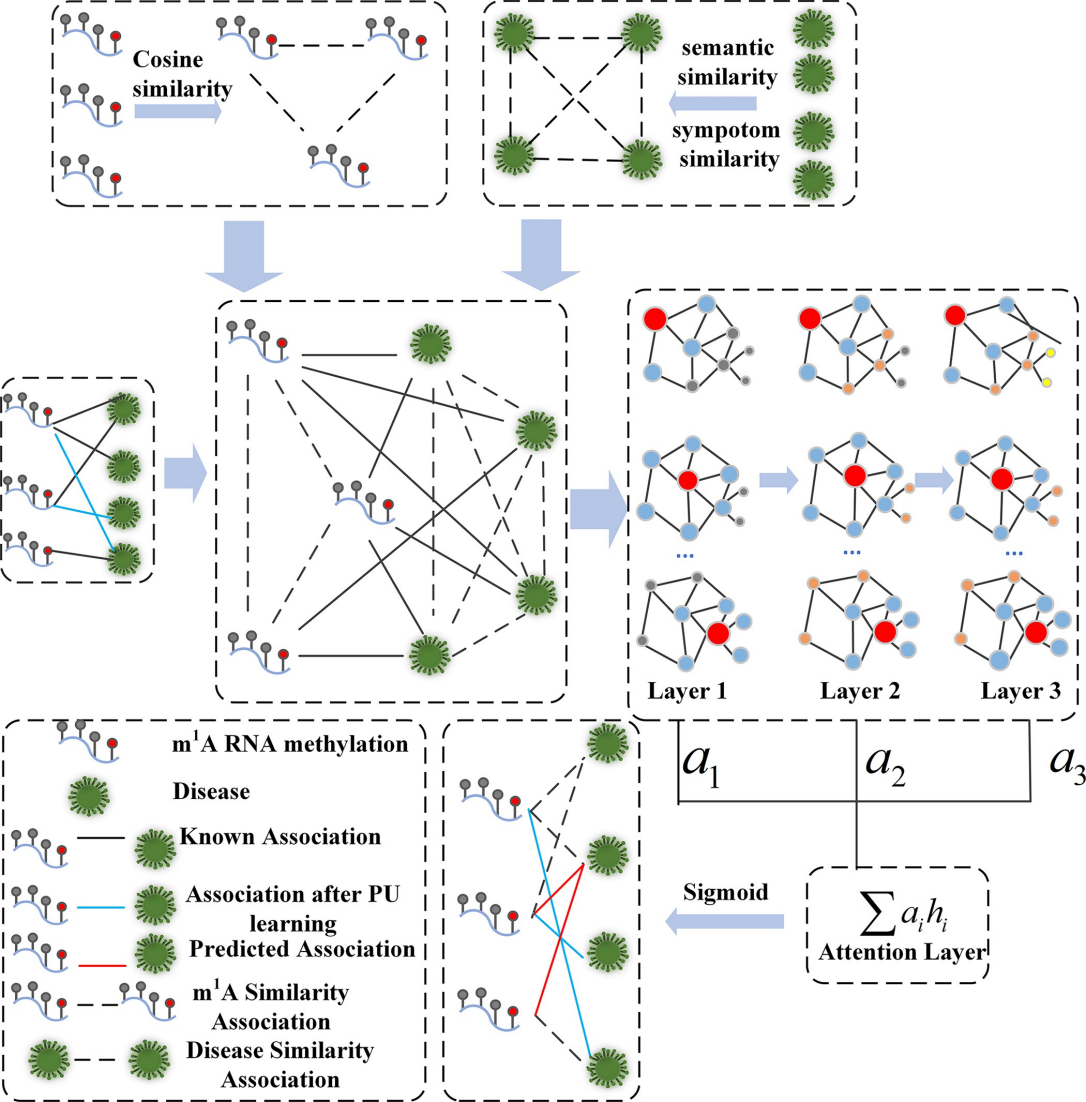
The flowchart of RMDGCN.

The whole flowchart of RMDGCN is shown in Fig 9.

### Dataset

The associations between m^1^As and diseases used by RMDGCN come from the RMVar database(http://rmvar.renlab.org) [31], which is specially used to collect RNA data of functional variants, and is designed to help reveal the potential function of RNA modified variants. In order to link RM-related variants with human genetic diseases, RMVar collected disease-related variants from GWAS queue and ClinVar database. In RMVar, we downloaded the dataset which is human related m^1^A information with “genetype = m^1^A” and “assembly = hg38”, including 62285 mutation related m^1^A methylation modification sites information. In the dataset, it includes modification sites location information, corresponding SNP information, tumor disease information, etc.

### Known associations between RNA Methylations and diseases

Due to the associations prediction of m^1^A modifications and diseases, we only focus on the sites information of m^1^A and the corresponding tumor diseases information. In the dataset we downloaded, it collected 62285 mutations related to m^1^A methylation modification sites and 424 diseases. In order to make the data have a higher confidence level, we reserve the modified sites with a high confidence level and diseases with corresponding DOID in the Disease ontology database (http://www.disease-ontology.org/) [32]. Finally, we obtained 3618 m^1^A modification sites, 116 diseases and 5100 associations. For convenience, we express the associations between m^1^A modification sites and diseases as a binary matrix *A*∈*R*^3618×116^. If there is interaction between m^1^A modification site *r*_*i*_ and disease *d*_*j*_, *A* (*i*, *j*) = 1; Otherwise, *A* (*i*, *j*) = 0.

### Similarity calculation

#### m^1^A RNA methylation similarity

In order to calculate the similarity of m^1^A modification, we first take the modification sites as the center, and lengthen the upstream and downstream by 250 bp. For each modification site, we finally obtain a sequence with the length of 501 bp based on the four AGCT bases. Word2vec [33] is a statistical method for learning word embedding. It can express a word into vector form quickly and effectively through the optimized training model according to the given corpus. It builds word embedding from the text corpus based on neural network training through two models, skip-gram and continuous Word Packet (CBOW). Skip-gram model can predict the surrounding words through the current words, while CBOW model can predict the current words through the context words. Both models focus on learning the context within the adjacent word window of a given word. We regard the length of three RNA nucleotides as an RNA word in the way of sliding window, and analyze their sequence content as an RNA corpus, and then use word2vec in the Gensim toolkit to learn the vector relationship of these RNA words, and finally generate a 100-dimensional feature vector for each sequence [34]. In the end, the sequences of all modified sites are converted into a data set *r* = *R*^*n*×*e*^, where *n* is the number of m^1^A methylation sites, and *e* is the dimensional of feature vector by word2vec.

Cosine similarity [35] is a measure of the difference between two individuals which uses the cosine value of the angle between two vectors in vector space. Compared with distance measurement, cosine similarity pays more attention to the difference between two vectors in the direction rather than the distance or the length. In order to measure the similarity of the two modification sites and construct the m^1^A-m^1^A similarity matrix, we use cosine similarity to calculate the similarity of the two m^1^A modification sites. The similarity between m^1^A *r*_*i*_ and m^1^A *r*_*j*_ can be defined as:

$$RRS(r_{i},r_{j})=\frac{r_{i}∙r_{j}}{\left\|r_{i}\|\times \|r_{j}\right\|}=\frac{\sum_{m=1}^{e}r_{i,m}\times r_{j,m}}{\sqrt{\sum_{m=1}^{e}{\left(r_{i,m}\right)}^{2}}\times \sqrt{\sum_{m=1}^{e}{\left(r_{j,m}\right)}^{2}}}$$ (2)

where *RRS*(*r*_*i*_, *r*_*j*_) is the similarity between m^1^A *r*_*i*_ and m^1^A *r*_*j*_, and the dimensions of similarity matrix *RRS* are 3618×3618, ‖•‖ denotes the L2 norm. Due to the range of cosine similarity being [–1,1], in order to keep the *RRS* similarity matrix within the range of [0,1], min-max normalization [36] is used to normalize the *RRS* similarity matrix.

### Disease similarity

RMDGCN constructs a disease-disease similarity network by calculating the semantic similarity and symptom similarity of diseases. The semantic similarity of diseases is calculated by using the DOID corresponding to diseases in the DOSE package of R language, and is recorded as *DOS*. According to the work of Zhou *et al*. [37], we can measure the disease similarity and build a symptom-based disease similarity network. Here, the symptom-based disease similarity matrix *DDS* is obtained from the disease symptom profile. Finally, the integrated disease similarity is regarded as the disease feature. The integrated disease similarity is calculated as follows:

$$DDS(d_{i},d_{j})=DOS(d_{i},d_{j})+(1-\lambda )DSS(d_{i},d_{j})$$ (3)

where *DOS*(*d*_*i*_, *d*_*j*_) is the semantic similarity between disease *d*_*i*_ and *d*_*j*_, *DSS*(*d*_*i*_, *d*_*j*_) is the symptom similarity between disease *d*_*i*_ and *d*_*j*_, *DDS*(*d*_*i*_, *d*_*j*_) is the integrated similarity between disease *d*_*i*_ and *d*_*j*_, *λ* is an adjusting parameter. The dimensions of similarity matrix *DDS* are 116×116.

### Positive-unlabeled learning

When the positive and negative samples are extremely unbalanced and the number of negative samples is far more than the positive samples, the effect of the model is very poor. In other words, the performance of the algorithm depends largely on a large number of known m^1^A modification sites and diseases associations. However, currently the known m^1^A modification sites and diseases associations are few, which means that the adjacency matrix *A* contains a large number of 0 elements. This will affect the performance of the model to a certain extent. In order to reduce the impact of sample imbalance caused by few positive samples on model performance, RMDGCN uses positive unlabeled learning to increase the number of positive samples to improve the predictive performance of the model.

**Fig 10:**
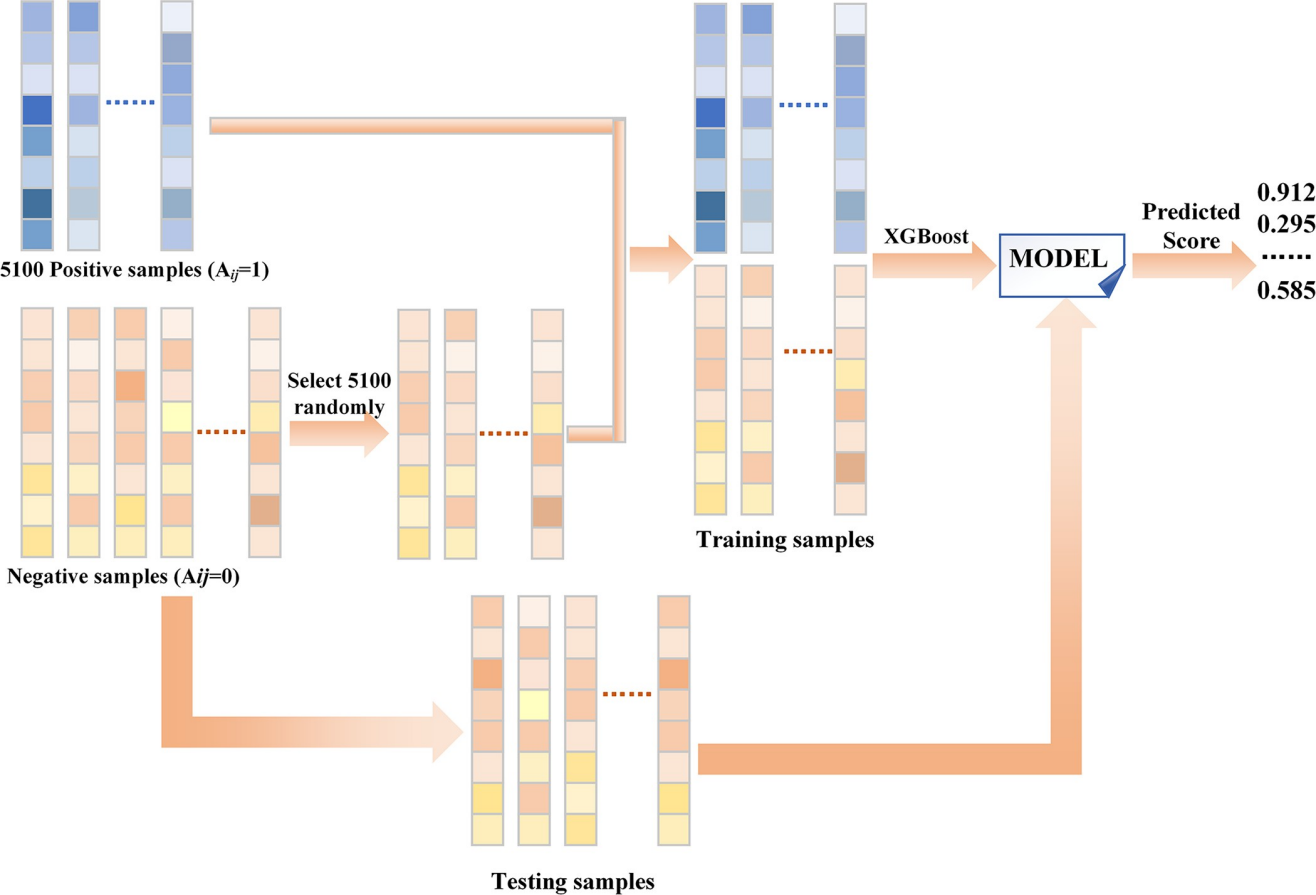
The flowchart of PU-learning.

We use *RRS* and *DOS* as features, randomly select the same number as the positive samples from the negative samples each time, and combine them with all positive samples to form training samples. Then the remaining negative samples are predicted to get the prediction score by XGBoost [38]. The detailed process is shown in Fig 10. We repeat the process (See Fig 10) 100 times. After repeating the process 100 times, for each negative sample, the average score of all predicted results is taken as the final score of the negative sample, while the score of the positive sample is still 1. Sample labels are confirmed based on a given threshold. If the score is greater than the threshold, then $\tilde{A}(i,j)=1$; otherwise, $\tilde{A}(i,j)=0$. Thus, a new adjacency matrix $\tilde{A}$ is constructed.

### Heterogeneous networks construction

Based on *RRS*, *DDS* and the new adjacency matrix $\tilde{A}$ after PU learning, the adjacency matrix representing heterogeneous networks is defined as follows:

$$X=\left(\begin{array}{cc} RRS & \tilde{A}\\ {\tilde{A}}^{T} & DDS \end{array}\right)$$ (4)

where *X* is the m^1^A-disease heterogeneous matrix, *RRS* is the similarity matrix between sites, *DDS* is the similarity matrix between diseases and diseases, $\tilde{A}$ is the adjacency matrix after PU learning, and ${\tilde{A}}^{T}$ is the transposition of $\tilde{A}$.

In order to construct a low dimensional representation of the relationships between m^1^As and diseases using GCN, we introduced penalty factors *α* and *β* on heterogeneous graph *X* to control the contribution of similarity in GCN propagation, and deployed GCN to combine node similarity and m^1^A-disease associations information.


$$G=\left(\begin{array}{cc} \alpha RRS & \tilde{A}\\ {\tilde{A}}^{T} & \beta DDS \end{array}\right)$$ (5)

### Graph convolution network with multilayer attention mechanism

Due to the ability of GCN to effectively extract spatial features of topology maps in non Euclidean structures, we choose GCN to extract features in the constructed heterogeneous network. There are two main types of graph convolutional neural networks, one based on spatial or vertex domains, and the other based on frequency or spectral domains. The essence of spatial graph convolution is to continuously aggregate the neighboring information of nodes, that is, directly accumulate the neighboring information of nodes to achieve graph convolution. Spectral domain based graph convolutional networks mainly rely on graph theory, correlated Fourier transform to achieve mutual conversion between spatial and spectral domains, and convolutional operation properties in spectral domains to complete the research of topological graph properties. In spectral-based graph neural networks, graphs are assumed to be undirected graphs, and one robust mathematical representation of undirected graphs is the regularized graph Laplacian matrix:

$$L=I_{n}-D^{-\frac{1}{2}}AD^{-\frac{1}{2}}$$ (6)

where *A* is the adjacency matrix of the graph, *D* is the diagonal matrix and

$$D_{ii}=\sum_{j}A(i,j)$$ (7)

Regularized graph Laplacian matrix has the property of being real symmetry semi-positive definite. Using this property, the regularized Laplacian matrix can be decomposed into

$$L=U\Lambda U^{T}$$ (8)

where $U=\{\vec{u_{1}},\vec{u_{2}},\ldots ,\vec{u_{n}}\in R^{N\times N}\}$, *U* is a matrix consisting of the eigenvectors of *L*, and *Λ* is a diagonal matrix, and the values on the diagonal matrix are the eigenvalues of *L*. The eigenvectors of the regularized Laplacian matrix form a set of orthogonal bases.

In graph signal processing, the signal *x*∈*R*^*N*^ of a graph is an eigenvector consisting of individual nodes of the graph, with *x*_*i*_ is thus defined as

$$F(x)=U^{T}x$$ (9)

The inverse Fourier transform is:

$$F^{-1}(\hat{x})=U^{T}\hat{x}$$ (10)

where $\hat{x}$ is the result of the Fourier transform.

In processing graphs, the discrete form of the Fourier transform is used. Since the Laplace matrix, after spectral decomposition, yields *n* linearly independent eigenvectors that form a set of orthogonal bases in space, the eigenvectors of the normalized Laplace matrix operator form the basis of the graph Fourier transform. The graph Fourier transform projects the signal of the input graph into the orthogonal space, which is equivalent to representing any vector defined on the graph as a linear combination of the eigenvectors of the Laplace matrix.

Specifically, given a network with the corresponding adjacency matrix *G*, the hierarchical propagation rule of the GCN is:

$$H^{\left(l+1\right)}=f(H^{\left(l\right)},G)=\sigma (D^{-\frac{1}{2}}GD^{-\frac{1}{2}}H^{\left(l\right)}W^{\left(l\right)})$$ (11)

where *H*^(*l*)^ is the embedding of the node at layer *l*, *D* = *diag*(∑_*j*_ *G*_*ij*_) is the degree matrix of *G*, *W*^(*l*)^ is the layer-specific trainable weight matrix, *σ*(•) is the nonlinear activation function, and $D^{-\frac{1}{2}}GD^{-\frac{1}{2}}$ is the normalized adjacency matrix. The left multiplication of the normalized adjacency matrix by the feature matrix is the aggregation process of neighbors, and then the right multiplication of *W* is the feature weighted summation process, and finally the nonlinear transformation is performed by the activation function.

In addition, considering that the contributions of different embeddings at different levels are inconsistent, we introduce attention mechanisms to combine these embeddings and ultimately obtain methylation modifications and disease embeddings:

$$\left[\begin{array}{c} Z_{R}\\ Z_{D} \end{array}\right]=\sum a_{l}H^{l}$$ (12)

where Z_*R*_ is the final m^1^A methylation modification embedding, Z_*D*_ is the final disease embedding, a is the weight of each layer.

**Fig 11:**
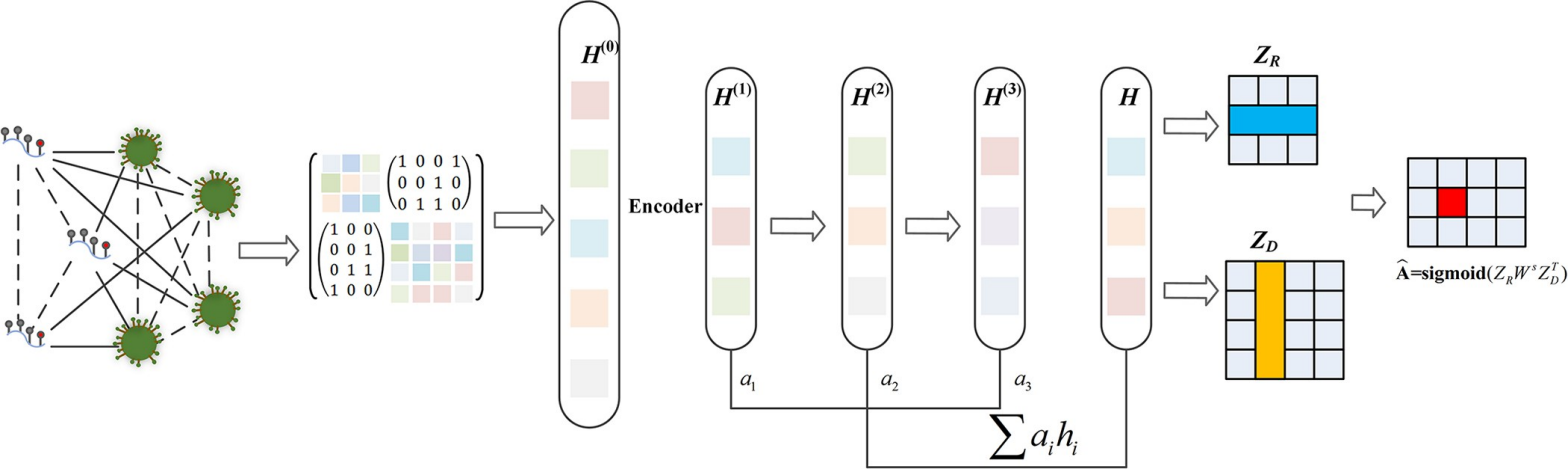
The detailed procedures of using GCN to predict the associations between m^1^As and diseases.

After the encoder processing, we obtain the potential feature vectors of m^1^As and diseases. Then we construct the m^1^A-disease network based on the potential feature vector Z_*R*_ and Z_*D*_, and expressed the predicted probability of the associations as follow:

$$\hat{A}=sigmoid(Z_{R}W^{s}Z_{D}^{T})$$ (13)

where $\hat{A}$ is the reconstructed adjacency matrix, in which the element represents the predicted score of relationship between the *i*th m^1^A and the *j*th disease. *W*^*s*^ is a trainable weight matrix. The detailed procedures of using GCN with multilayer attention mechanism to predict the associations between m^1^As and diseases are shown in Fig 11.

### Model training

RMDGCN consists of a multi-source heterogeneous network construction module and a GCN module. We obtained relevant information on the associations between m^1^As and diseases, using known associations as positive samples and unknown associations as negative samples. We divide it into five parts, with four parts serving as the training set and the rest part as the testing set.

Then we use the weighted cross entropy as the loss function:

$$L=-(\vartheta \sum_{\mathrm{i,j}}y_{ij}log\hat{y_{\mathrm{ij}}}+\sum_{\mathrm{i,j}}\left(1-y_{ij})log(1-\hat{y_{\mathrm{ij}}})\right)$$ (14)

where *ij* represents the relationship pair between the *i*th m^1^A and the *j*th disease, *y*_*ij*_ represents the true label of the relationship between the *i*th m^1^A and the *j*th disease, $\hat{y_{\mathrm{ij}}}$ represents the predictive score of the relationship between the *i*th m^1^A and the *j*th disease, and *ϑ* represents the cross entropy weight, which equals to the ratio of negative samples to positive samples.

To minimize the loss function, we use the Xavier method to initialize all the trainable weight matrices, and use the Adam optimizer [39] to minimize the loss function. The Adam optimizer can update the weights of the network according to the training data. We added a discard layer to avoid overfitting to achieve regularization effect [40]. In addition, we improve the convergence effect by cycling the learning rate [41] between the maximum and minimum values.

## Supporting information

S1 Data The information for 3618 m^1^A methylation sites. (XLSX)

S2 Data The known associations between m^1^A methylation sites and diseases. (XLSX)

S3 Data The top 500 candidate m^1^A methylation sites related to brease cancer by RMDGCN. (XLSX)

S1 Table The DOID for 116 diseases. (XLSX)

S2 Table The 150 host genes by GO analysis. (XLSX)
